# New Flexible Channels for Room Temperature Tunneling Field Effect Transistors

**DOI:** 10.1038/srep20293

**Published:** 2016-02-05

**Authors:** Boyi Hao, Anjana Asthana, Paniz Khanmohammadi Hazaveh, Paul L. Bergstrom, Douglas Banyai, Madhusudan A. Savaikar, John A. Jaszczak, Yoke Khin Yap

**Affiliations:** 1 Department of Physics, Michigan Technological University, Houghton, MI 49931, USA; 2 Department of Materials Science and Engineering, Michigan Technological University, Houghton, MI 49931, USA; 3 Department of Electrical and Computer Engineering, Michigan Technological University, Houghton, MI 49931, USA

## Abstract

Tunneling field effect transistors (TFETs) have been proposed to overcome the fundamental issues of Si based transistors, such as short channel effect, finite leakage current, and high contact resistance. Unfortunately, most if not all TFETs are operational only at cryogenic temperatures. Here we report that iron (Fe) quantum dots functionalized boron nitride nanotubes (QDs-BNNTs) can be used as the flexible tunneling channels of TFETs at room temperatures. The electrical insulating BNNTs are used as the one-dimensional (1D) substrates to confine the uniform formation of Fe QDs on their surface as the flexible tunneling channel. Consistent semiconductor-like transport behaviors under various bending conditions are detected by scanning tunneling spectroscopy in a transmission electron microscopy system (*in-situ* STM-TEM). As suggested by computer simulation, the uniform distribution of Fe QDs enable an averaging effect on the possible electron tunneling pathways, which is responsible for the consistent transport properties that are not sensitive to bending.

Carbon nanotubes (CNTs)^1^ and graphene^2^,^3^ have attracted tremendous research interest for applications in electronic devices. The highly flexible mechanical nature of CNTs and graphene has also enabled their applications in wearable devices^4^,^5^,^6^,^7^. Unfortunately, digital switches based on CNTs are limited by the uncontrollable chirality of as-grown single walled CNTs. Likewise the use of graphene in digital switches is prohibited by its zero band gap nature. On the other hand, the structurally analogous boron nitride nanotubes (BNNTs)^5^,^6^,^7^,^8^,^9^,^10^,^11^ and boron nitride nanosheets (BNNSs)^9^,^10^,^12^ have drawn increasing attention due to their extraordinary mechanical properties^13^,^14^,^15^, and highly flexible nanotubular structures^16^,^17^,^18^,^19^. However, pure BNNTs and BNNSs have a wide band gap (insulating materials)^20^,^21^, and thus are not applicable as the conduction channels of electronic devices. Significant efforts have been proposed to tune the electronic properties of boron nitride nanostructures, for example BNNTs, including doping with carbon^22^ or fluorine^23^, or applying giant Stark effect^24^, etc. However, all these attempts have not lead to any electronic devices.

Recently, we have shown that BNNTs functionalized with a one-dimensional (1D) array of gold (Au) quantum dots on their outer surfaces (QDs-BNNTs) could be used as the tunneling channels for tunneling field effect transistors (TFETs) at room temperature^25^,^26^. Results indicate that these TFETs are functional without involving any semiconducting properties as BNNTs are insulators and the QDs are metallic. In this case, BNNTs serve as the one-dimensional (1D) substrates to confine the gold QDs into a linear tunneling channel that conduct current only if sufficient bias voltages are applied. Because of the uniform insulating nature of BNNTs, QDs-BNNTs can be used in digital switches without the need of chirality sorting (as is needed for CNT devices). Band gap creation as required for graphene is also not needed for QDs-BNNTs. This means that switches based on QDs-BNNTs represent a novel class of electronics without the use of semiconducting materials. Thus these TFETs could avoid many of the fundamental scaling issues of Si FETs, including short channel effects, undesired heating due to finite current leakage, and high contact resistance with the metallic electrodes. These features make QDs-BNNTs a promising novel class of materials for future electronics with minimal issues of leakage current, overheating, and contact resistance. As supported by our computer simulations, these TFETs are operated according to the principles of single electron transistors (SETs).

There are several underlying properties of QDs-BNNTs that remain to be clarified. In particular, can QDs-BNNTs be used in flexible TFETs? Can other metallic QDs be applicable? To address these questions, we have prepared BNNTs functionalized with iron (Fe) nanoparticles to form Fe QDs-BNNTs. The room temperature tunneling behavior of these Fe QDs-BNNTs is then characterized by an *in situ* scanning tunneling microscopy and transmission electron microscopy (STM-TEM) technique. Results indicate that the tunneling behaviors of Fe QDs-BNNTs resembled the reported properties of gold QDs-BNNTs. Furthermore, *in situ* current-voltage (I-V) characteristics of Fe QDs-BNNTs are found to be resilient, even when the QDs-BNNTs are bent on the STM-TEM stage. These experimental results are analyzed and interpreted with the assistance of theoretical modeling and simulation. This extraordinary behavior suggests that Fe QDs-BNNTs are possible new materials for flexible TFETs without the limitations of traditional semiconductor devices.

Multi-walled BNNTs are grown in a conventional thermal chemical vapor deposition (CVD) system by the growth vapor trapping (GVT) approach^8^,^27^,^28^. As we have reported, the as-grown BNNTs are of high-quality with a uniform band gap of ~6 eV^8^,^27^. Figure 1(a) shows the long and clean morphologies of the as-grown BNNTs. These BNNTs are then coated with ~10 nm of iron film by a pulsed laser deposition (PLD) technique, followed by annealing in 300 sccm hydrogen at 700 °C for 30 minutes. This process leads to the formation of Fe QDs-BNNTs, as shown in Fig. 1(b). As shown in Fig. 1(c), transmission electron microscopy (TEM) suggests that small Fe QDs (3–9 nm) are formed around the whole surface of the BNNTs along with some additional QDs at one side of the BNNTs that are larger in diameter (10–15 nm). Raman spectra were collected as excited by a HeCd (λ = 325 nm) laser. As shown in Fig. 1(d), the characteristic Raman peak at ~1366 cm^−1^ corresponding to the *E*_*2g*_ in-plane vibrational mode are nearly identical between as-grown BNNTs and Fe QDs-BNNTs. This result suggests that the structural properties of BNNTs remain the same after the formation of Fe QDs. It is noted that the signal intensity of BNNTs is higher after the coating of Fe QDs. The enhanced Raman intensity is due to surface enhanced Raman scattering (SERS) induced by surface plasmons surrounding the iron nanoparticles^29^. However, the formation of Fe QDs-BNNTs are different from the formation of Au QDs-BNNTs as we previously reported^25^. In particular, whereas the Au QDs are present only along a line on the BNNTs, the Fe QDs are randomly dispersed around the entire surface the BNNTs (see Figure S1 for further details).

Electronic tunneling behavior of these Fe QDs-BNNTs is tested by using a STM-TEM system^16^,^30^,^31^. In this case, Fe QDs-BNNTs are transferred to the tip of a gold wire (250 μm in diameter) by mechanically scratching on the QDs-BNNT sample. The gold wire is then mounted to the piezoelectric stage as shown in Fig. 2(a). An individual Fe QDs-BNNT is then connected between the STM tip and the gold wire by driving by the piezoelectric stage. As shown in Fig. 2(b), a nonlinear I-V curve is detected with a clear Coulomb blockade feature at low bias voltages (off state). Current starts to flow when sufficiently high bias voltage is applied to initiate a cascade of electron tunneling events across the Fe QDs (on state). The turn-on voltage in this case is estimated as ~18 V [as marked by the red arrow in the inset of Fig. 2(b)]. This switching behavior is very well reproducible for all tested samples (see another example in Figure S2). In addition, we also verified that the detected current is due to tunneling across the Fe QDs as current is not detected when the QDs are removed (see supporting information and Figure S3). Such a semiconductor-like transport behavior is unexpected as both BNNTs and Fe QDs are not semiconductors.

Next, we evaluate the transport properties of the tunneling channel. We consider the Fe QDs-BNNT as a semiconductor-like channel, such that its transport behavior can be modeled as if it were a metal-semiconductor-metal (M-S-M) element^16^,^32^,^33^,^34^. From the M-S-M theory, the total current (*I*) near the turn-on stage (*V* ~ 18 V) is dominated by the reversed Schottky barrier in the intermediate bias regime. The total current can be expressed from 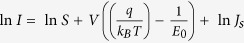, where *S* is the contact area, *J* is the current density through a Schottky barrier, *k*_*B*_ is the Boltzmann constant, *J*_*s*_ is the slowly varying function of the applied bias, and *E*_*o*_ is a function that depends on the carrier density; *i.e.* 
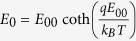
, where 
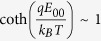
 and 
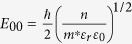
. Here, *ħ* is the Planck constant divided by 2π, *q* is the elemental charge, *n* is the carrier density, *m^∗^* is an effective electron mass (for our case, electron mass in vacuum), *ε_r_* is the relative permittivity (for vacuum in our case), and *ε_0_* is the vacuum permittivity. The ln *I* versus *V* plot gives an approximately straight line with a slope of 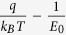 and an intercept of *ln SJs* on the y-axis, as shown in Fig. 2(c). This means, 
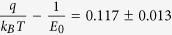
 V^–1^, such that *E*_*o*_, *E*_*oo*_, and *n* can be obtained. In this way, the electron mobility, μ, can be calculated by using the relation, 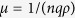 where *ρ* is the resistivity in the linear regime (at a bias voltage range of 22–32V) of the I-V curve shown in Fig. 2(b). All the calculated parameters are summarized in Table 1. Finally, we have also estimated the local density of states (LDOS) of the transport channel in Fig. 2(d). As shown, the normalized differential conductance [(*dI/dV*)/(*I/V*)] is plotted as a function of the bias voltage. The plot indicates two bands at positive and negative bias voltages, corresponding to the valance band and conduction band, respectively. The range of the relatively smooth conductance near the Fermi level (between the peaks on both sides) gives an estimate band gap of ~6.0 eV. Combining the transport properties in Table 1 and Fig. 2(d), the semiconductor-like behaviors of the Fe QDs-BNNT are much like a wide band gap semiconductor.

Next, we have evaluated the transport properties of Fe QDs-BNNTs under various bending conditions. To do so, the piezoelectric stage was used to gradually increase the mechanical strain on the connected Fe QDs-BNNT by pushing the Au wire toward the STM tip. TEM images of the bent QDs-BNNT are shown in Fig. 3(a–c) where the curvatures, *C* are 0 μm^−1^, 0.34 μm^−1^, and 1.69 μm^−1^ (corresponding to an estimated bending angle of 0°, 15°, and 75°, respectively). Here *C* = 1/*R*, where *R* is the arc radius of the bent QDs-BNNT.

Then the I-V transport properties of this QDs-BNNT are recorded. As shown in Fig. 3(d), bending of the QDs-BNNT does not dramatically change the transport properties. This signifies that QDs-BNNTs are promising tunneling channels for flexible TFETs. This observation may be explained as follows. For a linear QD chain^25^,^26^,^35^, as shown in Fig. 3(e) (left), electron tunneling will occur along the chain in an electric field. However, for a random QD chain shown in Fig. 3(e) (middle), electron tunneling will occur along a stochastic path defined by the greatest tunneling probabilities, as schematically illustrated in the figure. Depending on the details of the QD arrangement, the tunneling may take place among one or a few possible “optimal” pathways (dominant conducting paths)^26^ that are facilitated by the lower tunneling barriers and island charging energies. This is likely to be determined by relatively smaller QD separations in the random distribution. For the case of a bent Fe QDs-BNNT with Fe QDs distributed all around the BNNT, as illustrated in Fig. 3(e) (right), there will be both a compressed surface and a stretched surface. As compared to QDs located on the unbent surface, the inter-dot separations become smaller at the compressed surface and become larger at the stretched surface. However, since the QDs are distributed all around the BNNT only a small fraction of the QDs is located at the most compressed and stretched surfaces, thus most QD separations will be relatively unchanged. Thus the QD separations and the number of QDs involved in the tunneling process should, on average, be nearly the same for the bent and unbent cases. Therefore, the effect of bending should not significantly change the collected tunneling current across the device. Furthermore, the randomly distributed QDs will allow flexibility of electron tunneling at various alternative pathways when bending is introduced to different degrees. Such an averaging effect offered by the randomly distributed QDs is an interesting feature for using Fe QDs-BNNTs as the tunneling channel in future flexible and wearable TFETs.

It would be interesting to understand the carrier mobility of the devices. However, as we have reported for Au QDs-BNNTs, our back gate configuration is not ideal for such an investigation where only moderate gate effect was demonstrated^25^. Therefore we plan to study these topics with an alternative gate configuration for both Fe and Au QDs-BNNTs in the future. It is also noted that Fe has higher melting temperature than Au and will allow the formation of distributed QDs on the whole surface of BNNTs. The full surface distribution of Fe QDs is the key property for flexible tunneling channels discussed here. However, the annealing leads to larger separation gaps between Fe QDs, as compared to the case of Au QDs. Therefore, the source drain current is lower due to higher tunneling resistance.

Computer simulations were performed to qualitatively understand the relatively robust transport properties of Fe QDs-BNNTs (see Supplementary Information and Figure S4, S5, and S6), following the methodology described by Savaikar, *et al.*^26^,^35^. Instead of simulating QDs with randomized spatial distributions around the entire two-dimensional tubular surface as shown in Fig. 1c, we simulate the effects of bending on a linear array of Fe QDs distributed on one side of the BNNT with positive and negative curvatures. This simplifies the study to investigate the most extreme effects of bending on a simple hypothetical dominant conducting path. In this model, the Fe QDs are distributed along a linear chain, and are isolated from each other by vacuum with source and drain electrodes on either end. In brief, the modeled system consists of a 775 nm long linear array of 49 islands (QDs) distributed between two electrodes as shown in Fig. 3e (left). In order to simulate that the islands are sitting on one side of a BNNT surface, the islands are aligned such that they all touch a hypothetical BNNT tube of radius 20 nm, but the effects of the BNNT are otherwise ignored. The island radii were chosen randomly in a range between 3.2 and 9.9 nm, and the inter-island separation is varied randomly from 1.1 nm to 4.9 nm. These value ranges are estimated based on the discussion of Fig. 1c.

The I-V characteristics of the device were first simulated for the chain of QDs bent at a wide range of curvatures, *C* ranging from 0 to ±3.38 μm^−1^ (corresponding to total bend angles ranging from 0 to ±150° for our relatively short chain). As shown in Fig. 4(a), the tunneling current across the array of QDs increases when the chain is bent upward and as the interisland separations decrease. According to theory (Supporting Information), the probability of an electron tunneling through a junction, 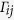 depends on the tunneling resistance (*R*_*ij*_), temperature (*T*) and the change in the free energy (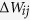) upon tunneling. For a given source-drain bias, 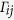 is most sensitive to *R*_*ij*_. As 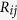 depends on the junction width/separation between QDs (*d*_*ij*_), the effective tunneling barrier height (*Φ*_eff_), and the Fermi energy (*E*_*F*_) of iron (*E*_*F*_ is constant for the chain, but *Φ*_eff_ decreases with increasing potential difference across a junction), the tunneling probability and current will increase with the decrease of the junction width (*d*_*ij*_). Thus the simulated current and its dependence on curvature shown in Fig. 4(a) are understandable. Likewise for negative curvatures, wherein the spacing between QDs increase, the current decreases relative to the unbent case.

Next we simulated the averaging effect of the random distribution of QDs on the overall tunneling current. As illustrated in the inset of Fig. 4(b), a linear QD chain with both positive and negative curvatures (bend angle of ±30°) is simulated. As expected, the tunneling current for the chain bent at +30° is higher than the unbent device due to the decrease of QD separations, whereas, the tunneling current for the chain bent at −30° is lower, due to the QD separations being increased. Figure 4(b) also shows that the average current, taken as the simple average of the currents in the ±30° bend cases, is nearly the same as the current flow in the straight (unbent) QD chain. The average results do show a slightly higher current since the effect of decreasing island separations has a stronger increase in current than the comparable decrease in current for a comparable increase in island separations. As noted above, these two simulated cases represent the extremes among the actual island-separation changes expected for the QD-BNNTs with QDs randomly distributed all around the BNNT. The simulated results suggest that when averaging effects are taken into account as for the case of a random QD chain, the change of QD separations on the compressed and stretched surfaces will not result in significant changes in the overall tunneling current of the device. Even if QDs deposited on the compressed and stretched surfaces are involved, their positive and negative contribution will be largely cancelled or averaged out, and lead to near zero net effect.

In conclusion, we have demonstrated a novel class of flexible channels for TFETs. This was created by Fe QDs-BNNTs where iron nanoparticles are randomly distributed on the surface of the insulating BNNTs. We show that tunneling current across Fe QDs-BNNTs is switchable, with obvious on and off states. This switching behavior is intact and reproducible at bending angles up to 75°. Theoretical simulation supports our explanation where the contribution of QDs deposited on the bent surfaces is not significant, in particular after an averaging effect occurred on the randomly distributed QDs. Therefore, Fe QDs-BNNTs is a new class of functional materials for flexible switches without involving any semiconducting nature. This novel class of flexible electronics would potentially have a low heating effect, low contact resistant issue, and ignorable short channel effect, due to the quantum tunneling nature of the devices.

## Methods

### Fe QDs-BNNTs fabrication

Multi-walled BNNTs are prepared in a conventional thermal chemical vapor deposition system (LINDBERG/BLUE M model) by the growth vapor trapping approach. These BNNTs are then coated with ~10 nm of iron film (measured by quartz monitor) by PLD technique (fourth harmonic generation of a Nd:YAG laser at wavelength =266 nm, and pulse duration =5 ns), followed by annealing in 300 sccm hydrogen ambient at 700 °C for 30 minutes.

### Characterization

The surface morphology of Fe QDs-BNNTs is characterized by Hitachi S-4700 FESEM and JEOL JEM 4000FX TEM (operated at 200 kV). The structural property of Fe QDs-BNNTs is characterized by Jobin-Yvon LabRAM HR800 Raman Spectrometer. All the electrical measurement was carried out in a STM-TEM holder (Nanofactory Instruments) in the Jeol JEM 4000FX TEM system that operated at 200 kV.

## Additional Information

**How to cite this article**: Hao, B. *et al.* New Flexible Channels for Room Temperature Tunneling Field Effect Transistors. *Sci. Rep.* **6**, 20293; doi: 10.1038/srep20293 (2016).

**Reprints and permission** information is available online at http://www.nature.com/reprints.

## Supplementary Material

Supplementary Information

## Figures and Tables

**Figure 1:**
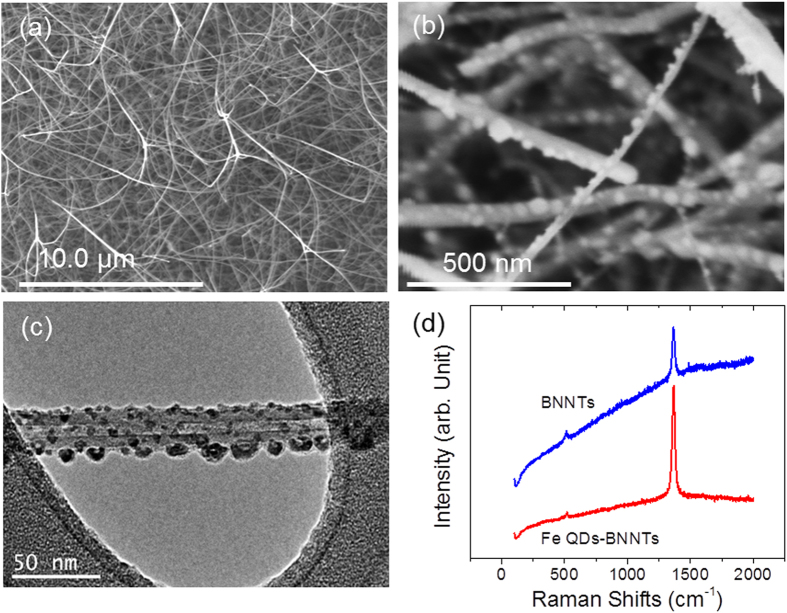
Iron quantum dots functionalized boron nitride nanotubes (Fe QDs-BNNTs). Images of Fe QDs-BNNTs as revealed by (**a,b**) scanning electron microscopy, and (**c**) transmission electron microscopy. (**d**) The Raman spectra of BNNTs and Fe QDs-BNNTs are nearly identical, signifying that the BNNT structural properties are retained after QD deposition.

**Figure 2:**
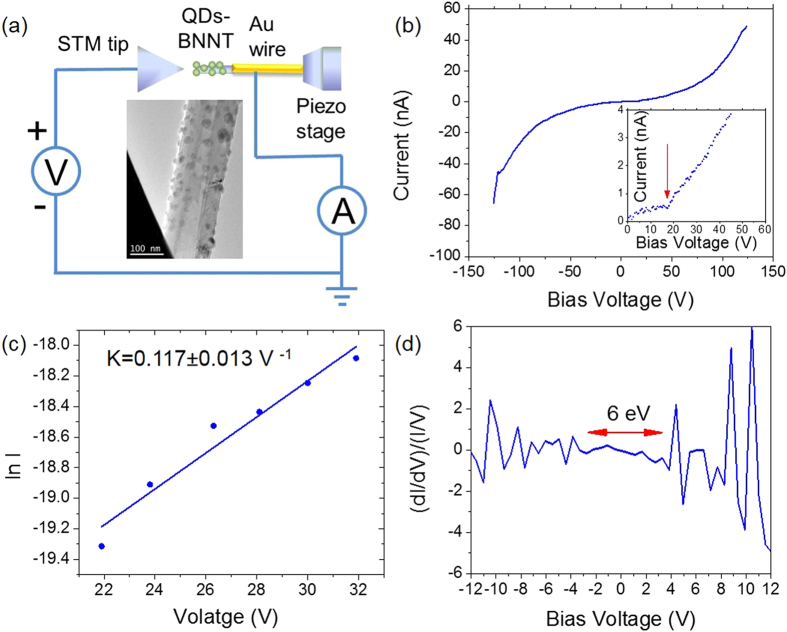
Electronic properties of a Fe QDs-BNNT. (**a**) Schematic for current-voltage (I-V) measurement of individual Fe QDs-BNNTs by a STM-TEM stage. (**b**) The typical switching behavior of a Fe QDs-BNNT with a clear turn-on voltage (the red arrow in the inset). (**c**) Fitting of ln I versus V for the intermediate bias regime of Fig. 3b with a slope of K. (**d**) The normalized differential conductance plot for the corresponding I-V behavior.

**Figure 3:**
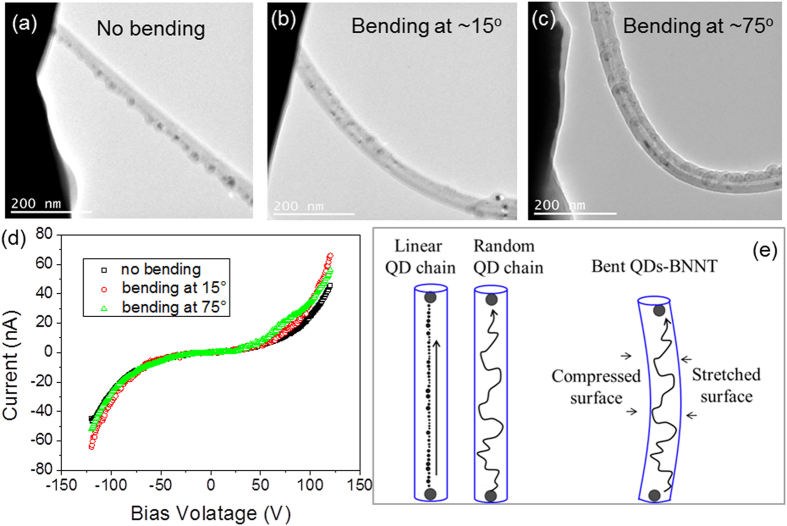
Invariant transport properties of a Fe QDs-BNNT. (**a–c**) TEM images of a Fe QDs-BNNT at various bending angles. (**d**) Current-voltage (I-V) behavior of the QDs-BNNT as measured by the STM-TEM stage at various bending angles. (**e**) Schematic drawing of current pathways on QDs-BNNTs with linear (left) and random (middle) QD chains. Schematic drawing of the current pathway for a bent QDs-BNNT with random QD chain (right).

**Figure 4:**
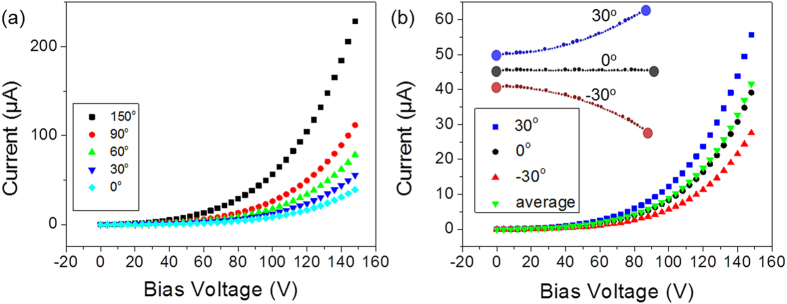
Simulated transport properties of a Fe QDs-BNNT. (**a**) Simulated I-V characteristics of a QDs-BNNT at various bending angles. (**b**) Simulated I-V characteristics of a QDs-BNNT at bending angles of 0° and ±30° (zero curvature and ± 0.67 μm^−1^) and the average I-V curve for cases bent at ±30°.

**Table 1:** Parametric Electronic properties of Fe QDs-BNNTs.

Resistance[GΩ]	Resistivity[Ω·cm]	Carrier concentration [cm^−3^]	Mobility [cm^2^/V·s]	Band gap [eV]
1.27 ± 0.01	9.97	1.94 × 10^24^	3.2 × 10^−7^	6.0
