# Supplementary material for: New Flexible Channels for Room Temperature Tunneling Field Effect Transistors
Source: Sci Rep. 2016 Feb 5;6:20293. doi: 10.1038/srep20293 (PMC4742867; doi:10.1038/srep20293)
Supplement: Supplementary Information [file srep20293-s1.doc]

**Supplementary Information**

**New Flexible Channels for Room Temperature Tunneling Field Effect Transistors**

Boyi Hao^1^, Anjana Asthana^2^, Paniz Khanmohammadi Hazaveh^3^, Paul L. Bergstrom^3^, Douglas Banyai^1^, Madhusudan A. Savaikar^1^, John A. Jaszczak^1^, Yoke Khin Yap^1*^

^1^Department of Physics, Michigan Technological University, Houghton, MI 49931, USA.

^2^Department of Material science and engineering, Michigan Technological University, Houghton, MI 49931, USA. ^3^Department of Electrical engineering, Michigan Technological University, Houghton, MI 49931, USA.

^*^To whom correspondence should be addressed. E-mail: [ykyap@mtu.edu](mailto:ykyap@mtu.edu)

1. **Formation of Fe QDs-BNNTs versus formation of Au QDs-BNNTs**


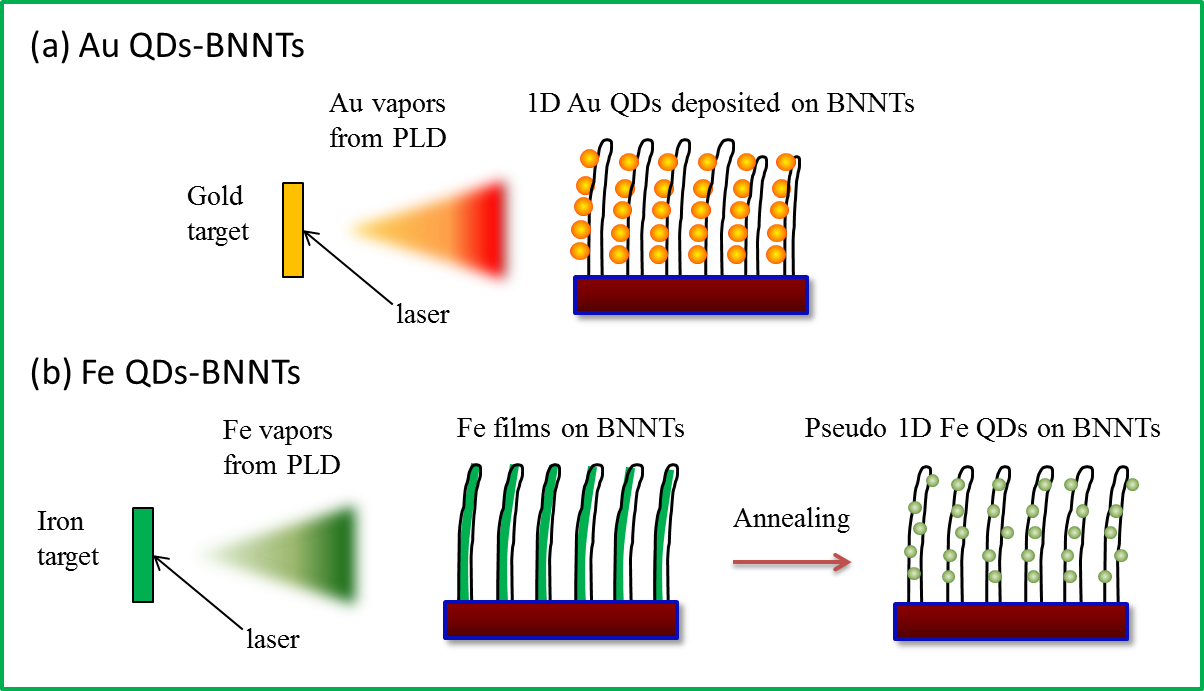


Figure S1. Schematic drawing on the formation of (a) gold QDs-BNNTs and (b) Fe QDs-BNNTs.

The formation of gold QDs and Fe QDs on BNNTs are different. Gold QDs can be formed by PLD coating without annealing, as shown in Figure S1a. These gold QDs are deposited on one side of the BNNTs that is facing the laser plume. Because of the higher melting and evaporation temperatures of iron, Fe QDs formation requires annealing. This annealing results in a more uniform distribution of Fe QDs around the whole surface of the BNNTs (Figure S1b). As also suggested by SEM and TEM images in Figure 1, Fe QDs are larger in diameter (10-15nm) on the side of the BNNTs that faced the laser plume, in addition to the smaller Fe QDs (~3-9 nm) that are distributed elsewhere. The distances between the smaller Fe QDs are also smaller (a few nm) compared to those between the bigger QDs (as large as ~10 nm). Because of the smaller inter-dot separations, the smaller Fe QDs are most likely to be those responsible for electron tunneling of TFETs, as consistent with our earlier-reported devices [1, 2].

1. **Switching of Fe QDs-BNNTs**

Another example of current switching on a Fe QDs-BNNT is shown in Figure S2.


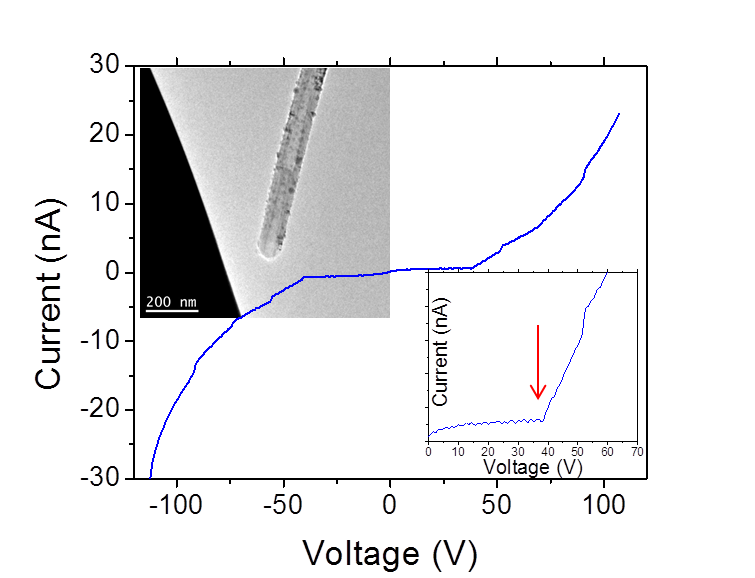


Figure S2. Current-voltage characteristic of an individual Fe QDs-BNNTs as probed by a STM-TEM probe. The turn-on threshold bias is indicted by the red arrow in the inset.

The fabrication of Fe QDs-BNNTs is based on thermal annealing of Fe film with specific thickness on BNNTs. Ideally, we would be able to obtain reproducible I-V characteristic on different samples by keeping the same coating and annealing condition. However, we still see variation of turn on voltages due to the actual length of QDs-BNNT that is attached on the STM-TEM probe. For example, the turn-on voltage in Figure S2 is higher than the example in Figure 2(b).

1. **Function of Fe QDs on swiching**

A bunch of Fe QDs-BNNTs were sampled on an Au wire. The same sample was then dipped into hydrochloric acid (HCl, 36.5-38.0%) to remove the FE QDs (Figure S3a and S3b). As suggested by TEM images, all of the iron nanoparticles were removed after etching as shown in the inset of Figure S2c. As shown, switching behavior is not detected after etching. This result verifies that QDs are required to facilitate electron tunneling across the QDs-BNNTs. The insulating nature of BNNTs remain intact as BNNTs are inert to acid treatment.


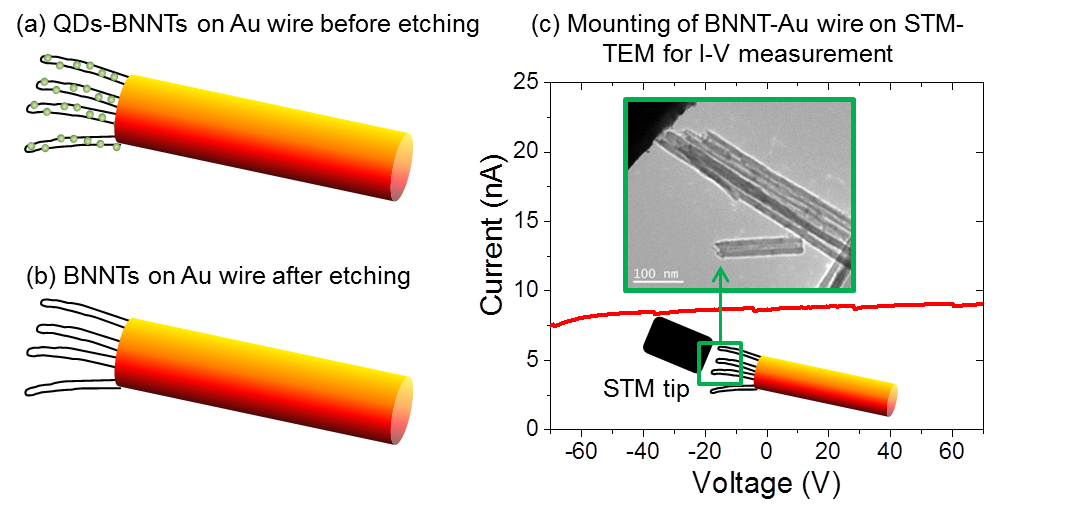


Figure S3. Schematic of a bunch of QDs-BNNTs attached on the Au wire (a) before and (b) after etching. (c) I-V characteristic of these etched QDs-BNNTs show no switching behavior from the etched tubes (inset).

1. **Theory**

Modeling of this system is based on orthodox theory for tunneling transport onto a quantum dot [3], and implemented for arbitrary system geometries utilizing the Kinetic Monte Carlo methodology, as outlined in Savaikar, et al. [4]. The IV characteristic of a one-dimensional (1D) chain of Iron nano-islands on an insulating substrate (bent or not bent) is a function of the chain-length and temperature. Under high bias conditions, as in this experiment, temperature has a minimal effect and the IV characteristics are non-Ohmic, and Coulomb staircase structures will not be observed. For high biases, the simulation starts from zero volts and increases in steps of 4 volts up to 150 volts.

The transport channel consist of a one dimensional chain of iron quantum dots, which are isolated from each other by vacuum, with source and drain electrodes on either end. Applying voltage between the electrodes, which are playing source and drain roles, causes a potential difference between neighboring islands. Tunneling between the neighboring islands occurs when the potential difference (
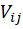
 ) is high enough.

Tunneling between two islands occurs when the free energy of the system decreases.
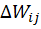
 is the change in the free energy of the system due to the transition (
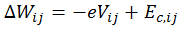
). Charging energy (
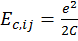
) is the energy required for an electron to tunnel through the barrier between two coupled islands, and *e* is the electron charge.

Probability of an electron tunneling through a junction depends on the tunneling resistance (
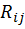
), temperature (*T*) and the change in the free energy (
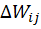
). Tunneling resistance depends upon the junction width (*d_ij_*), barrier height, fermi energy (4.5ev for iron) and the geometry of the junctions. The dependence on the junction width is confirmed with the simulations of the bent device (as the device bends, the junction length changes so the tunneling rate and the current changes as well). The height of the potential barrier is the second term, which impacts the tunneling resistance. The height of the barrier depends on the work functions of the corresponding islands. It has been considered that the barrier’s height is constant across the width of the junction
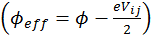
 . Iron work function is 4.5ev [3].

Following Savaikar et al. (2013), the tunneling rates for a tunneling transition between any pair of islands *i,j* is calculated from:


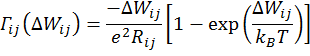


So when the free energy decreases the electron can tunnel but depending on the tunneling resistance the rate is different.

In previous studies, the distances between the islands are given as an input. Assuming that the device is bent, the islands distance will change which will affect the tunneling resistance and tunneling rates. When the degree of bending is entered, the model will calculate the new distances between the islands. The potential drop across a junction between two islands depends on the charge state of the two islands and the system’s capacitances. Writing the equation, *Q = CV*, for each junction is used to build a *C* matrix. The charge state of the islands changes when a tunneling event occurs, which results in an update in the charge state of the overall system. The tunneling rates of the junctions are calculated based on the orthodox theory. Kinetic Monte Carlo method is used to randomly choose which tunneling event will occur. Using Monte Carlo method tunneling with higher probability is chosen more often than the less probable ones. When the conditions for tunneling are satisfied, the charge state of the system is updated and the voltage drop across the junctions are recalculated using *Q = CV*. The tunneling rates are updated after each event, and the process will be repeated until the deviation in the total current is less than 5 percent for a given bias condition [4].

The IV characteristic of the device for high bias source-drain voltages (from 0 to 150 volts) and different curvatures (from -3.5 to 5.1 μm^-1^) was simulated using Matlab. Figures S4-S6 show the final results for the system. Figure S4 shows the currents for different curvatures. More details of the variation is observed in Figure S5, which is zoomed in. In Figure S6, the semi log based plot of the IV characteristic is shown.


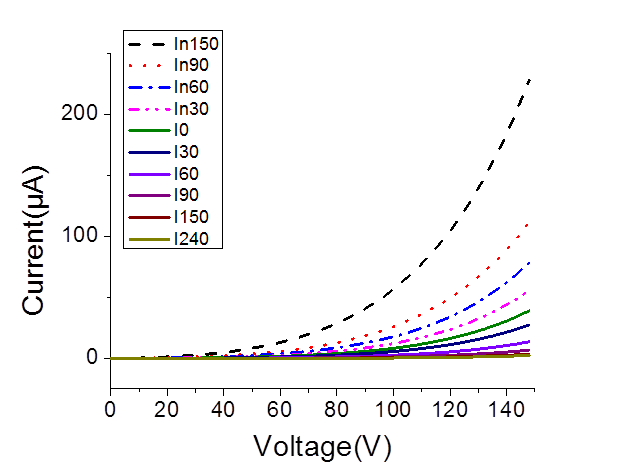


Figure S4. The IV characteristic of the bent multi-island tunneling device in high bias regime. Each color shows a different curvature (from -3.5 to 5.1 μm^-1^).


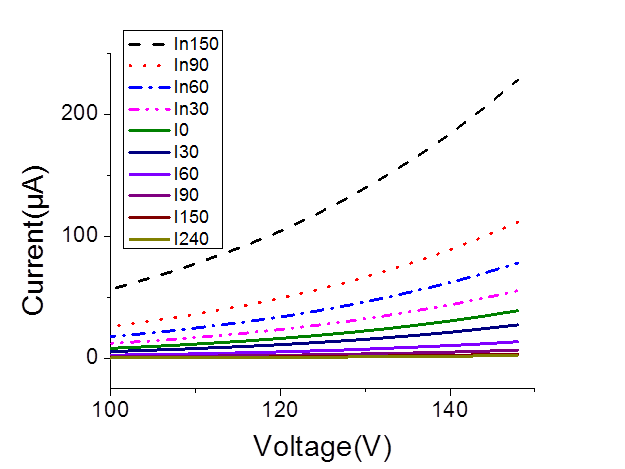


Figure S5.The IV characteristic of the bent multi-island tunneling device in high bias regime. Each color shows a different Curvatures (from -3.5 to 5.1 μm^-1^).


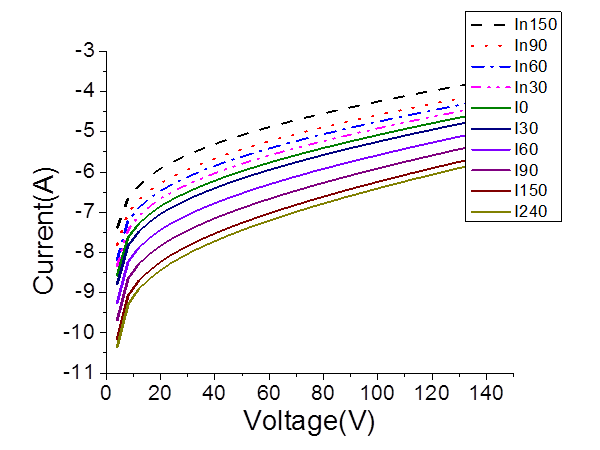


Figure S6. The semi-log plot for IV characteristic of the bent multi-island tunneling device in high bias regime. Each color shows a different Curvatures (from -3.5 to 5.1 μm^-1^).

When the system bends the junction length changes and correspondingly changes the IV characteristic of the system. When the curvature is positive (bending downward), the resulting current decreases. When the curvature is negative (upward), the junction length decreases and the current increases. This increase in current is expected as the tunneling resistance depends exponentially on this length (*d_ij_*). The tunneling resistance increase and the tunneling rate decreases so the current decreases. The simulation result for the bent system supports this hypothesis. It can be observed in Figure S4 that as the device bends up the current decreases, and as it bends down the current increases. Matching precisely the magnitude of the quantitative results with the experimental results is not germane to the analysis, since the trends in the IV characteristic behaviors are consistent. In the experimental system, the junction width and quantum island diameter have a wider variation than was modeled, which results in a current that diverges in magnitude from the simulation, but retains the same trend and profile.

There is a limitation for high bias transport that has not been considered by this modeling effort. The individual junction bias voltage must remain below the magnitude of the effective work function approximation that is used for the junction. The model will not converge for negative effective work functions. In this case, for 47 junctions and a maximum 150 V applied bias at the terminals, the individual effective work functions did not exceed this limitation.

**References:**

[1] C. H. Lee, S. Qin, M. A. Savaikar, J. Wang, B. Hao, D. Zhang, D. Banyai, J. A. Jaszczak, K. W. Clark, J. C. Idrobo, A. Li, Y. K. Yap. Room-temperature tunneling behavior of boron nitride nanotubes functionalized with gold quantum dots. *Advanced Materials* **25,** 4544 (2013).

[2] J. A. Jaszczak, M. A. Savaikar, D. R. Banyai, B. Hao, D. Zhang, P. L. Bergstrom,; A.-P. Li, J.-C. Idrobo, Y. K. Yap. Simulation of charge ttransport in disordered assemblies of metallic nano-islands: application to boron-nitride nanotubes functionalized with gold quantum dots. *MRS Online Proceeding Library* **1700,** 17 (2014).

[3] K.k. Likharev. Single-electron Devices and Their Applications. *Proceedings of the IEEE* 87.4 (1999): 606-32.

[4] M. A. Savaikar, D. Banyai, P. L. Bergstrom, J. A. Jaszczak. Simulation of Charge Transport in Multi-island Tunneling Devices: Application to Disordered One-dimensional Systems at Low and High Biases. *Journal of Applied Physics* 114.11 (2013): 114504.
